# Red‐light flashing pens and seizures in children

**DOI:** 10.1111/dmcn.70001

**Published:** 2025-10-03

**Authors:** Simone Gasparini, Alice Dainelli, Francesca Piras, Simona Balestrini, Renzo Guerrini

**Affiliations:** ^1^ Neuroscience and Human Genetics Department Meyer Children's Hospital IRCCS Florence Italy; ^2^ Department of Neuroscience, Pharmacology, and Child Health University of Florence Florence Italy

## Abstract

Photosensitive epilepsy is the most common form of stimulus‐induced epilepsy. We describe two unrelated females aged 9 years and 10 years, with prolonged photic‐induced absence seizures triggered by a commercially available red‐light flashing pen popular among children. Both patients underwent clinical assessment and electroencephalography (EEG) with intermittent photic stimulation. Their EEGs showed a generalized photoparoxysmal response to intermittent photic stimulation. Exposure to the pen, performed in the 9‐year‐old, elicited a powerful photoparoxysmal discharge. Both patients were successfully treated with valproic acid, preventative measures, and avoidance of known triggers. These observations highlight the risk posed by poorly regulated flashing lights in consumer products. Despite past efforts to regulate visual stimuli in video games and media, current regulatory frameworks do not adequately address emerging light‐emitting diode‐based technologies, leaving vulnerable populations exposed to preventable risks. There is a pressing need for updated safety standards to prevent seizure‐provoking stimuli, especially in products marketed to children.

## Graphical abstract

Guidelines exist to limit seizure‐provoking visual stimuli. However, existing recommendations are not universally applied, and poorly regulated flickering lights are easily encountered in consumer products. Two girls experienced prolonged absence seizures triggered by a red‐light flickering pen. Their EEGs showed a photoparoxysmal response to either intermittent photic stimulation, or direct pen exposure, or both. After valproic acid treatment was introduced and preventative measures illustrated, no further seizure have occurred. We emphasize the need for internationally applied updated safety standards.

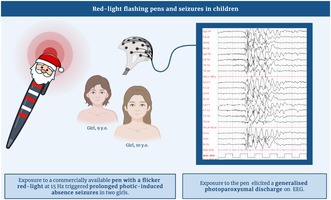

Plain language summary: https://onlinelibrary.wiley.com/doi/10.1111/dmcn.70045

**Abbreviation**
LED: light‐emitting diode

**What this paper adds**

- Common consumer devices for children may deliver seizure‐inducing visual stimuli.
- Light‐emitting diode regulation with international reach for child‐accessible products is urgently needed.

Photosensitive epilepsy, the most common form of stimulus‐induced epilepsy, is characterized by seizures triggered by specific visual stimuli such as light flashes, flashing lights, colour transitions, or repetitive patterns.^1^, ^2^ In patients with photosensitive epilepsy, intermittent photic stimulation during electroencephalogram (EEG) typically evokes a photoparoxysmal response, defined as an abnormal discharge provoked by intermittent light stimulation.^3^ The estimated prevalence of photosensitive epilepsy is approximately 1 in 4 000 people in the general population, with the highest incidence between 7 years and 20 years of age, and clear female predominance.^2^ About 9% to 15% of individuals with generalized epilepsy and 0.9% to 3% of those with focal epilepsy exhibit photosensitivity.^2^

Although the pathophysiological mechanisms underlying photosensitive epilepsy remain poorly understood, available evidence points to disruptions in contrast gain control and heightened responsiveness to specific spatial and temporal frequencies of visual input.^4^, ^5^, ^6^ Seizure‐provoking stimuli typically involve bright flashes (> 20 candelas/m^2) at 3 Hz to 60 Hz (with peak sensitivity at 15 Hz–20 Hz), covering at least 10% to 25% of the visual field and certain visual parameters such as red flashes (wavelengths of 660 nm–720 nm) and alternating patterns like stripes.^7^, ^8^

In recent decades, light‐induced seizures have been increasingly associated with digital media, including television content (e.g. the Pokémon incident of 1997), video games, and social media videos.^2^, ^9^, ^10^ Here, we report two unrelated females who experienced light‐induced seizures triggered by exposure to the same type of commercially available red‐light flashing pen.

## STANDARD PROTOCOL APPROVALS, REGISTRATIONS, AND PATIENT CONSENTS

Written informed consent for participation in the research and publication of the results was obtained from the guardians (parents) of both patients.

## CASE REPORT

### Patient 1

Patient 1 is a female with typical developmental skills who at age 9 years experienced two episodes of impaired awareness with unresponsiveness and motionless staring, lasting several minutes. The first episode occurred during exposure to multiple strobe light reflections while on a spinning ride in a fairground, and the second was triggered by a flashing red‐light pen flashing at around 15 Hz. An EEG performed after the first seizure showed normal background activity and a generalized photoparoxysmal response. Brain magnetic resonance imaging (MRI) was normal.

**FIGURE 1:**
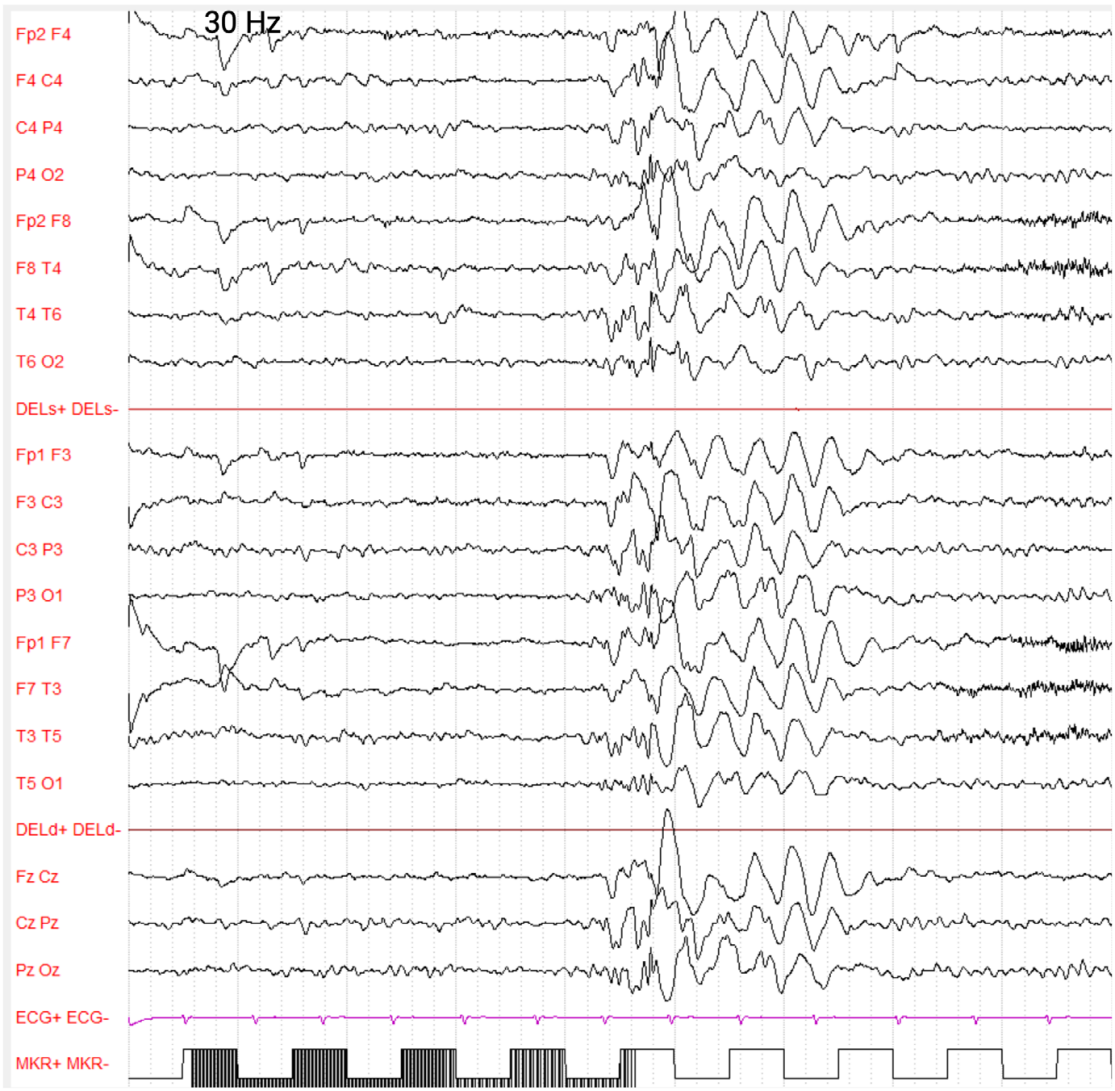
EEG recording at first clinical evaluation during 30 Hz intermittent photic stimulation in Patient 1. During intermittent photic stimulation, generalized polyspike‐wave discharges are observed at 30 Hz. Standardized 10–20 system montage. Amplitude: 130uV/cm, 20 second/page.

**FIGURE 2:**
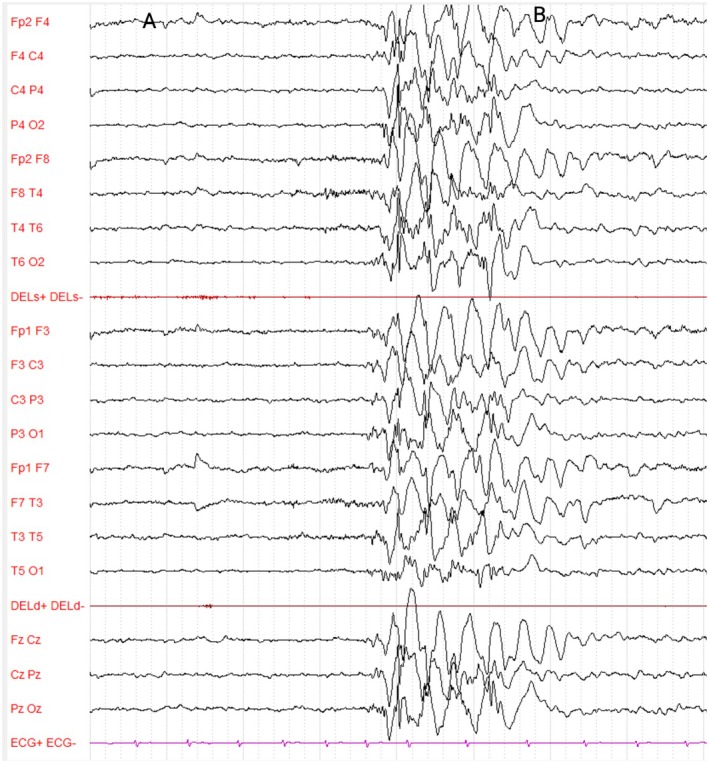
EEG recording at first clinical evaluation during exposure to the flashing pen in Patient 1. During exposure to the flashing pen (Video S1), generalized polyspike‐wave complexes appear. (a) The patient starts staring at the flashing pen. (b) The technician withdraws the pen from the patient's view. Standardized 10–20 system montage. Amplitude: 130uV/cm, 20 second/page.

After the second seizure, an EEG recording with a dedicated protocol for visual sensitivity revealed a generalized photoparoxysmal response induced by intermittent photic stimulation at 9 Hz to 30 Hz (Figure 1), by exposure to both vertical and horizontal stripe patterns, and 3 seconds after staring at the flashing red‐light pen that had precipitated the seizure (Figure 2; Video S1). Based on clinical and EEG evidence, we classified this patient's seizures as prolonged photic‐induced absence seizures and initiated valproic acid at 300 mg/day. We also advised the use of blue‐tinted polarized lenses in environments with high light contrast. The patient has remained seizure free for 12 months after starting treatment. A follow‐up EEG under treatment showed a photoparoxysmal response with lowering of the upper threshold to 20 Hz. Determination of blood levels of the drug could not be obtained.

### Patient 2

Patient 2 is a 10‐year‐old female with typical developmental skills who, when aged 9 years, experienced her first and only seizure triggered by exposure to the same flashing red‐light pen as Patient 1. This episode was characterized by impaired awareness with unresponsiveness and motionless staring, lasting about 3 minutes and followed by drowsiness. An EEG, performed 2 days later, showed normal background activity, a generalized photoparoxysmal response at 30 Hz, and generalized discharges of polyspikes during sleep and upon awakening. Brain MRI was normal.

**FIGURE 3:**
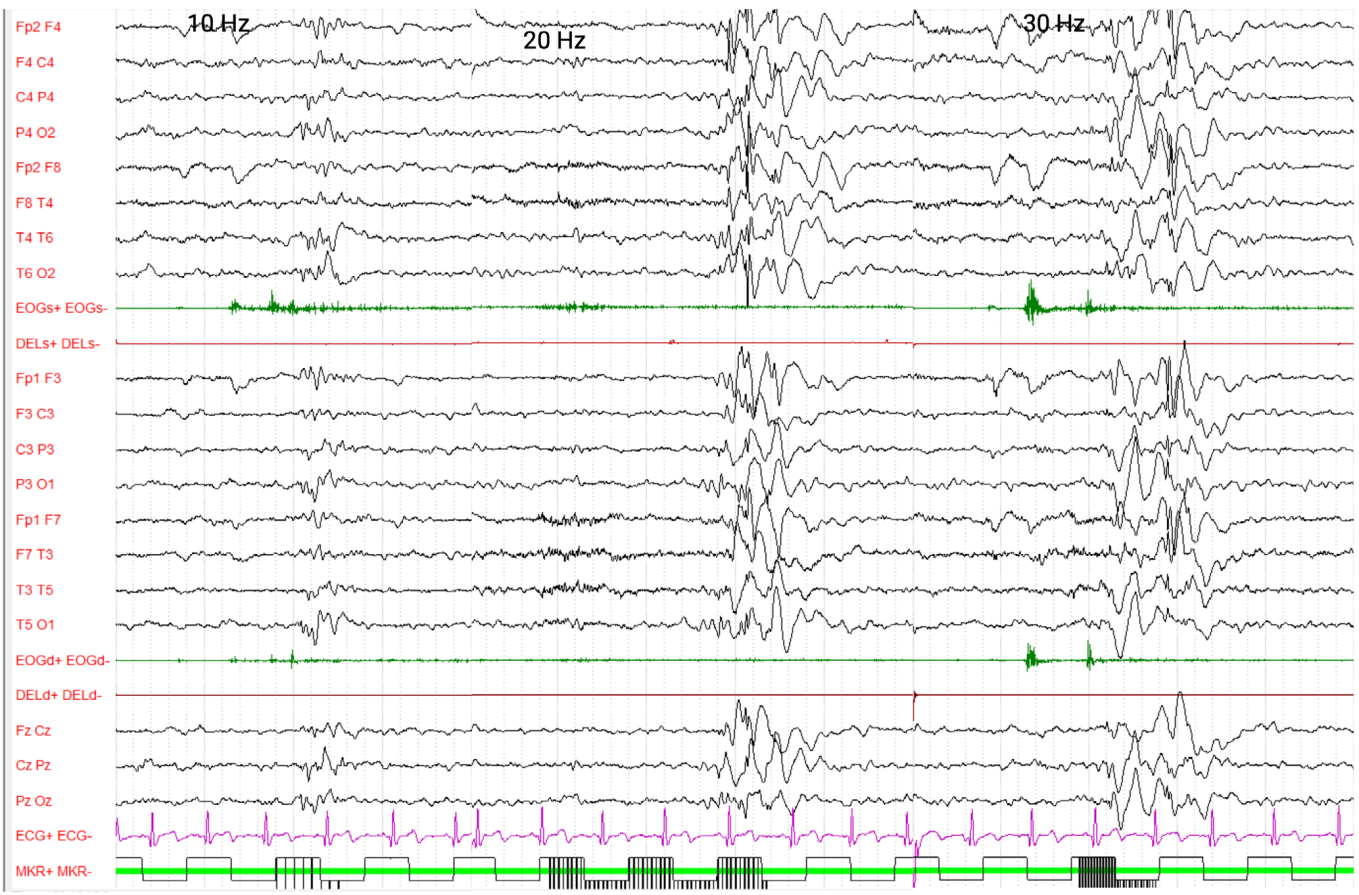
EEG recording at first clinical evaluation during intermittent photic stimulation in Patient 2. Intermittent photic stimulation elicits 1‐ to 3‐second‐long discharges of Polyspikes and slow waves complexes at frequencies between 10 Hz, 20 Hz, and 30 Hz. Standardized 10–20 system montage. Amplitude: 130 uV/cm, 20 second/page.

Two months later, an EEG recording with a dedicated protocol for visual sensitivity revealed that the photoparoxysmal response was precipitated by intermittent photic stimulation between 10 Hz and 30 Hz (Figure 3). Based on clinical and EEG evidence, we classified this patient's only seizure as a prolonged photic‐induced absence seizure, initiated 600 mg/day valproic acid, and advised the use of blue‐tinted polarized lenses in environments with high light contrast. A follow‐up EEG, 10 months later, showed an attenuated photoparoxysmal response, only occurring inconstantly at 25 Hz. Maintenance dose serum valproate level was 72 mg/L. No further seizures occurred during the 2‐year follow‐up.

Next generation sequencing analysis of 372 genes associated with epilepsy was unrevealing.

## DISCUSSION

The two observations presented here provide evidence that red‐light flashing pens, seemingly innocuous consumer gadgets, can provoke seizures in photosensitive individuals, even uncovering previously unrecognized epilepsy. Both patients exhibited good clinical and electrographic response to valproic acid and visual environmental adaptation.

The 1997 Pokémon incident, when a 6‐second‐long televised sequence of alternating red and blue lights flashing at 12 Hz provoked seizures in hundreds of child viewers, paved the way for guidelines aimed at reducing the risk of seizures caused by broadcast material.^2^, ^8^ Originally developed in the UK and Japan, and later spread worldwide by the International Telecommunications Union, these guidelines prevent broadcasting specific flashes frequencies (>3 Hz) and areas (> 25% screen), and certain changes in luminance and colour, including certain types of moving patterns.^8^ The introduction of these regulations was associated with a reduction in seizures attributed to television broadcasts in Japan.^8^

The widespread adoption of video games and reports of seizure‐related incidents have prompted the development of accessibility guidelines for game developers.^11^ These guidelines include avoidance of flashing images that last for more than 5 seconds and instantaneous high change in brightness/contrast or to/from the colour red.^11^ Neither of these measures are respected by the flashing light‐emitting diode (LED) lighting pen in question.

It has been demonstrated that existing LED lighting technologies can produce flashes at frequencies capable of triggering biological responses in humans.^12^ To address this issue, strategies to reduce the unintended biological effects of LED lighting have been proposed.^12^ Despite current recommendations, recent episodes of seizures triggered by digital content on social media,^2^ as well as the two exemplar observations presented here, claim that efforts to prevent marketing of visual triggers that can precipitate seizures remain inconsistent.^2^ Red light has been associated with the strongest triggering effect among flashing light stimuli compared to other colours.^10^ Nevertheless, red flashing lights continue to appear in commercially available products.

Moreover, seizure risk warnings, such as those on the product leaflet of these pens, are often vague and difficult to locate, making them easily overlooked. Parents, educators, and even some clinicians may be unaware of the risks these devices pose. Trendy or novelty items with strong visual appeal can quickly gain popularity among children, heightening the risk of unintentional exposure. As a result, the paediatric population—the group most vulnerable to visually‐induced seizures—remains an unfortunate and frequent target of such hazardous products.

Our observations underscore the need for ongoing scientific attention and stricter jurisdictive awareness on the subject, particularly regarding LED flicker and flashes in commonly merchandized objects and devices to ensure the safety of at‐risk populations in increasingly technology‐driven environments.

## Supporting information


**Video S1:** Red‐light flashing pen. The video shows the red‐light flashing pen that triggered seizures in both patients

## Data Availability

No data sets were generated or analysed during the current study.
